# LILRA5 Functions to Induce ROS Production on Innate Immune Cells

**DOI:** 10.1002/eji.70079

**Published:** 2025-10-20

**Authors:** Zuyi Fu, Matevž Rumpret, Irina Kube‐Golovin, Mykola Lyndin, Vera Solntceva, Yuxi Zhao, Anastasia Konieva, Na Liu, Adrian T. Press, Stefanie B. Flohé, Michael Bauer, Gunther Wennemuth, Bernhard B Singer, Alex J. McCarthy

**Affiliations:** ^1^ Department of Infectious Diseases Centre for Bacterial Resistance Biology Section of Molecular Microbiology, Imperial College London London UK; ^2^ Department of Anatomy Medical Faculty University of Duisburg‐Essen Essen Germany; ^3^ Department of Pathology Academic and Research Medical Institute Sumy State University Sumy Ukraine; ^4^ Department of Medical Microbiology UMC Utrecht Utrecht The Netherlands; ^5^ Department of Anesthesiology and Intensive Care Medicine Jena University Hospital Jena Germany; ^6^ Center for Sepsis Control and Care Jena University Hospital Jena Germany; ^7^ Medical Faculty Friedrich‐Schiller‐University Jena Germany; ^8^ Department of Trauma Surgery University Hospital Essen Essen Germany

**Keywords:** activating receptor, infection, LILRA5, reactive oxygen species, sepsis

## Abstract

Activating immune receptors provides mechanisms for phagocytes to elicit important effector functions that promote the killing of microbes. Leukocyte immunoglobulin‐like receptor A5 (LILRA5), an orphan immune receptor expressed by human phagocytes and co‐localising with FcRγ, remains poorly characterised. To address this, we developed a highly specific anti‐LILRA5 monoclonal antibody that has agonistic properties. We show LILRA5 expression on naïve monocytes and neutrophils, and that ligation of LILRA5 stimulates ROS production. While increased *LILRA5* transcripts have been associated with sepsis, we also observed increased levels in patients with systemic infection but without sepsis complications. Ex vivo bacterial infection of whole blood did not alter surface LILRA5 expression, but LPS stimulation changed expression levels, indicating that surface LILRA5 expression is dynamic and likely regulated post‐transcriptionally, changing responses to different stimuli or over time. Soluble (s)LILRA5 was enhanced in sera from sepsis patients and in supernatants of monocytes that were LPS‐stimulated, indicating that shedding of LILRA5 from cell surfaces or that expression of sLILRA5 isoforms provides a mechanism to regulate surface LILRA5 expression levels. Finally, we show that altered surface LILRA5 expression influences LILRA5‐induced ROS production capacity. Thus, LILRA5 is a dynamically regulated activating receptor expressed on phagocytes that stimulates ROS production.

## Graphical abstract

Ligation of the activating LILRA5 receptor specifically stimulates ROS production by monocytes and neutrophils. Though *LILRA5* transcripts are upregulated upon immune stimulation, surface LILRA5 expressions are dynamically regulated. Enhanced soluble LILRA5 was observed during immune challenge, suggesting that dynamic regulation of LILRA5 modulates ROS induction and innate immune responses.

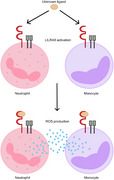

**Abbreviations**
DAMP: damage‐associated molecular pattern
DB: dynabeads
FcRγ: Fc receptor gamma‐chain
FCS: foetal calf serum
FcαR: Fc alpha receptor
FITC: fluorescein Isothiocyanate
GFP: green fluorescent protein
IFN‐γ: interferon gamma
IgG1: immunoglobulin G1
IL‐1β: interleukin‐1 beta
IL‐6: interleukin 6
ITAM: immunoreceptor tyrosine‐based activation motif
LAIR1: leukocyte‐associated immunoglobulin‐like receptor 1
LILRA2: leukocyte immunoglobulin‐like receptor A2
LILRA5: leukocyte immunoglobulin‐like receptor A5
LPS: lipopolysaccharide
mAb: monoclonal antibody
NFAT: nuclear factor of activated T cells
PAMP: pathogen‐associated molecular pattern
PBMCs: peripheral blood mononuclear cells
PE: phycoerythrin
ROS: reactive oxygen species
TNF‐α: tumour necrosis factor alpha

## 1 Introduction

Phagocytic immune cells such as neutrophils, monocytes and macrophages play a key role in the innate immune response to infection [1]. They can kill invading pathogens by engulfing them through phagocytosis, releasing antimicrobial molecules and generating reactive oxygen species (ROS). This is attributed to the expression of immune receptors on the cell surface that detect signs of infection. Given the importance of phagocytosis, degranulation and ROS generation, these processes can be activated via several innate immune receptors. This includes receptors signalling via immunoreceptor tyrosine‐based activation motif (ITAM) in their cytoplasmic tail [2, 3, 4] or those that co‐associate with the ITAM‐containing FcRγ [1, 5]. Though several innate immune receptors co‐associate with FcRγ [6], their capacity to activate phagocytes, induce degranulation and/or stimulate ROS production is less well understood.

Leukocyte immunoglobulin‐like receptor A5 (LILRA5, ILT11, LIR9, CD85f) is a member of the leukocyte immunoglobulin‐like receptor (LILR) family [7]. *LILRA5* transcripts are expressed by neutrophils, monocytes and macrophages [8, 9]. Whilst surface LILRA5 expression has been reported for monocytes and macrophages [8], the expression of LILRA5 on neutrophil surfaces remains unclear [10]. LILRA5 contains two extracellular immunoglobulin (Ig) domains, a transmembrane region for co‐localisation with the ITAM‐containing FcRγ and a short cytoplasmic tail [8]. It can also be expressed in a soluble form known as sLILRA5 [8, 9]. Though the ligands of LILRA5 are unknown [7, 11], cross‐linking LILRA5 on monocytes induces FcRγ association, ITAM phosphorylation in FcRγ and the release of pro‐inflammatory cytokines [8]. Mice with deletion of the *LILRA5* gene displayed an increased susceptibility to bacterial keratitis and a dysregulated inflammatory response [12].

Due to the scarcity of highly specific anti‐LILRA5 mAbs and no knowledge on endogenous and exogenous LILRA5 ligands [10], the expression and functions of LILRA5 are poorly elucidated. Recent reports have shown that *LILRA5* transcripts are significantly increased in human bacterial keratitis and in human sepsis [12, 13]. This is suggestive that LILRA5 has a role in microbial defence and could be a useful biomarker for rapid diagnosis of inflammation triggered by pathogens. In this study, we developed a highly specific antibody against LILRA5 as a tool to investigate expression and functions. We investigated *LILRA5* transcript levels and surface LILRA5 expression on phagocytes in health and disease, and assessed whether cross‐linking LILRA5 could trigger ROS production and phagocytosis.

## 2 Results

### 2.1 Development of LILRA5‐Specific mAb with Agonistic Properties

**FIGURE 1:**
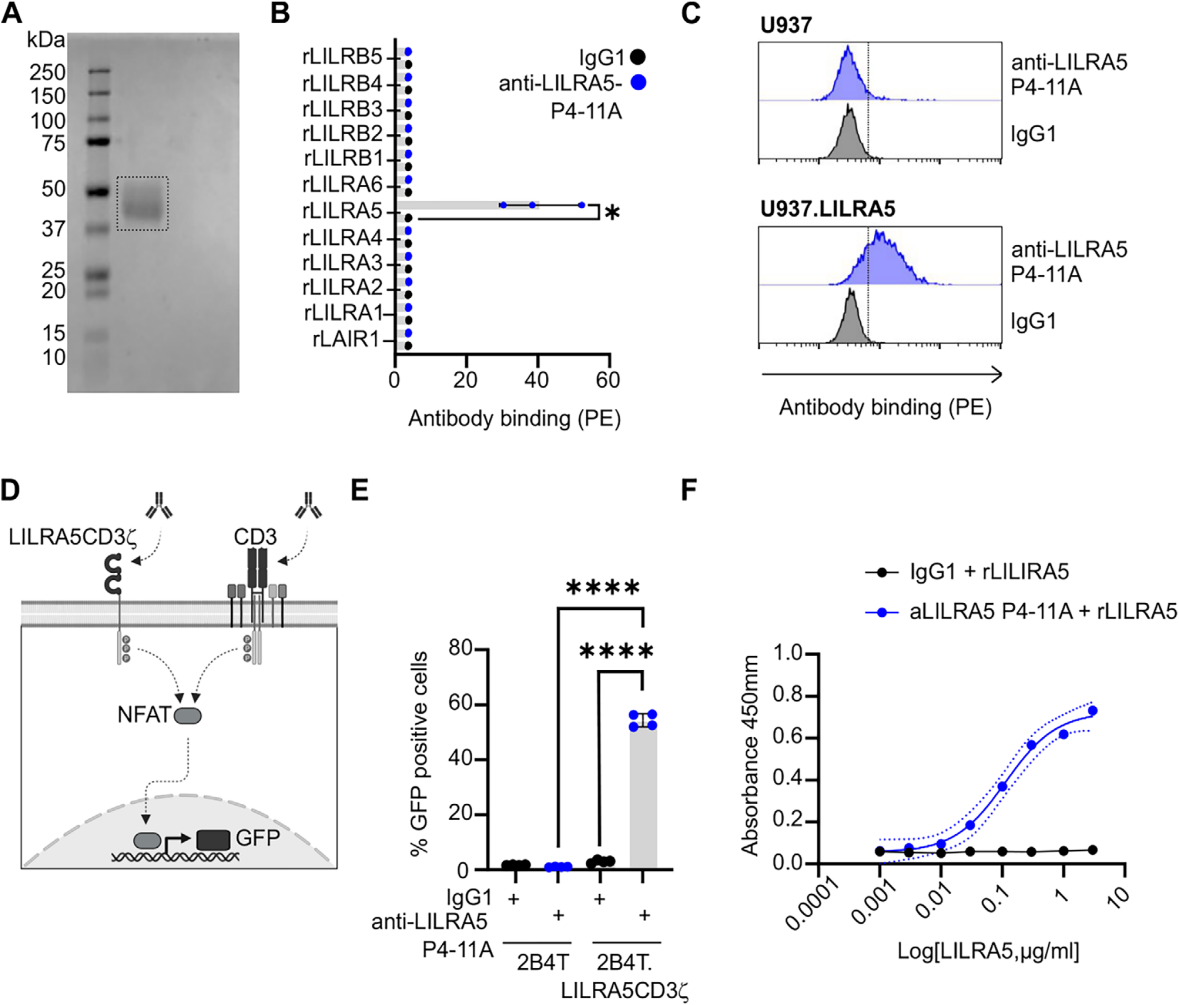
A highly specific anti‐LILRA5 antibody with cross‐linking capacity. (A) SDS‐PAGE analysis of rLILRA5‐His. (B) Binding of anti‐LILRA5 P4‐11A mAb to magnetic beads coated with rLILR or control protein, detected using anti‐IgG mAb and flow cytometric analysis. Mean ± SD of *n* = 3 independent experiments. Paired *t*‐test. (C) Binding of anti‐LILRA5 clone P4‐11A mAb to U937 cell lines, detected using anti‐IgG mAb and flow cytometric analysis. One representative experiment from *n* = 3 independent experiments. (D) A schematic of the LILRA5CD3ζ reporter 2B4 T cell line, expressing a surface protein composed of extracellular and transmembrane LILRA5 domains fused to the cytoplasmic tail of CD3ζ. Cross‐linking of CD3 or the fusion LILRA5CD3ζ protein induces ITAM phosphorylation, NFAT activation and GFP expression. (E) Reporter cells stimulated with anti‐CD3 mAb, anti‐LILRA5 P4‐11A mAb, or isotype control. Mean ± SD of *n* = 4 independent experiments. (F) Detection of rLILRA5‐His by ELISA using anti‐LILRA5 P4‐11A mAb or isotype control. Mean and SD of *n = 3* experiments. In all, *p < 0.05 and ****p < 0.0001.

First, recombinant (r)LILRA5 was (Figure 1A) used to immunise  BALB/c mice. Anti‐LILRA5 P4‐11A mAb was purified from a hybridoma derived from the fusion of myeloma NS1/0 cells with spleen cells. Anti‐LILRA5 P4‐11A mAb bound to magnetic beads coated with human rLILRA5, but not to any of the closely related human rLILR that are most likely to be cross‐reactive, nor to an immune receptor from a different family called LAIR1 (Figure 1B; Figure S1A,B). Furthermore, anti‐LILRA5 P4‐11A recognised rLILRA5 in a concentration‐dependent manner (Figure S1C), indicating high specificity for detecting LILRA5. Flow cytometry analysis also revealed enhanced binding of anti‐LILRA5 P4‐11A to the LILRA5+ U937 cells compared with control U937 cells (Figure 1C), revealing that anti‐LILRA5 P4‐11A can specifically detect cell surface‐expressed LILRA5.

As cross‐linking is crucial for studying mAb‐induced LILRA5 function, we tested whether anti‐LILRA5 P4‐11A could cross‐link and activate LILRA5 in a reporter cell model. We generated 2B4T NFAT‐GFP reporter cells that express the LILRA5CD3ζ fusion protein at the cell surface (Figure 1D), an established approach for identifying functional LILR ligands [14, 15, 16, 17]. Anti‐CD3 induced GFP expression in LILRA5 and control cells (Figure S2A), whilst rabbit polyclonal anti‐LILRA5 induced GFP expression in only the LILRA5 cells (Figure S2B). Notably, anti‐LILRA5 P4‐11A induced the expression of GFP in LILRA5CD3ζ‐expressing, but not control, 2B4T NFAT‐GFP cells (Figure 1E). Therefore, anti‐LILRA5 P4‐11A has agonistic properties for LILRA5 that can be utilised as a molecular tool to study LILRA5 functions.

Next, we investigated whether anti‐LILRA5 P4‐11A could be used as a capture antibody in an ELISA assay. We used rLILRA5‐His as a standard and rabbit anti‐LILRA5 polyclonal antibodies for detection. The ELISA detected rLILRA5‐His, but not rLAIR1‐His as a control protein, in a concentration‐dependent manner (Figure 1F). Thus, the anti‐LILRA5 P4‐11A antibody binds to human LILRA5, does not cross‐react with other LILRs and has agonistic properties.

### 2.2 LILRA5 is Expressed on the Surface of Human Neutrophils and Monocytes

**FIGURE 2:**
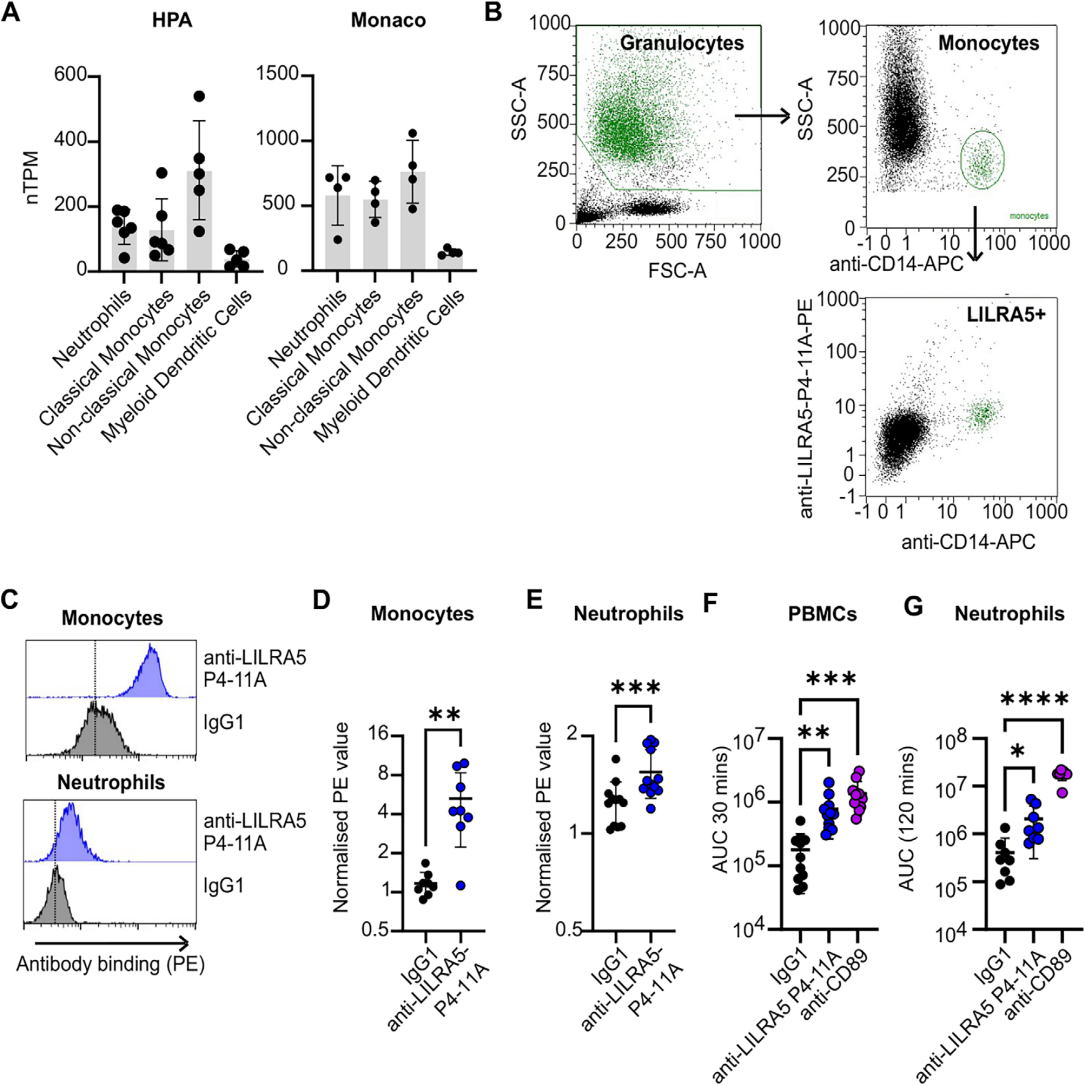
LILRA5 is expressed on human phagocytes and stimulates ROS production. (A) Normalised transcripts per million (nTPM) of *LILRA5* in immune cells from the HPA and Monaco datasets. Mean ± SD are shown. (B) Representative example showing the gating strategy used to identify monocytes in human whole blood using anti‐CD14 and anti‐LILRA5 (clone P4‐11A). A first gate was set on physical parameters of SSC‐A vs. FSC‐A, then on SSC‐A and SSC‐H to eliminate doublets, then monocytes and granulocytes were gated on CD14+ and CEACAM8+ (not shown), then on CD14+ events to identify monocytes. (C) Representative flow cytometry staining indicating LILRA5 expression in neutrophils and monocytes from a healthy donor. (D, E) Expression of LILRA5 on human monocytes (D, *n* = 8) and neutrophils (E, *n* = 12), determined using anti‐LILRA5 P4‐11A or isotype control. Paired *t*‐test. (F, G) Reactive oxygen species (ROS) production by PBMCs (F, *n* = 7) and neutrophils (G, *n* = 8). One‐way ANOVA of area under the curve (AUC) values, in all, ****p < 0.0001, **p < 0.01, and *p < 0.05.

Since LILRA5 protein levels on neutrophils were never reported [10], we analysed transcript expression from contemporary RNA‐seq data and protein expression using the anti‐LILRA5 P4‐11A. Analysis of RNA‐seq data showed that *LILRA5* transcript abundance was comparable between neutrophils and monocytes, but at a lower level on dendritic cells (DCs) (Figure 2A). PE‐conjugated anti‐LILRA5 P4‐11A bound to the surface of 95.58 ± 7.44 % of monocytes and 99.57 ± 0.47 % of neutrophils from *n* = 3 donors (Figure 2B; Figure S3A–C). Further analysis revealed significantly higher signals for anti‐LILRA5 P4‐11A compared with IgG1 for monocytes (5.28 ± 3.05 vs. 1.17 ± 0.25) (Figure 2C,D) and neutrophils (1.55 ± 0.26 vs. 1.24 ± 0.19) (Figure 2C,E). Notably, the surface LILRA5 expression was lower for neutrophils when compared with monocytes. Taken together, this reveals that naïve neutrophils and monocytes express comparable transcript levels of *LILRA5*, while monocytes express higher protein levels of LILRA5 on their cell surfaces.

### 2.3 Respiratory Burst Can be Stimulated Through LILRA5

Next, we investigated whether LILRA5 cross‐linking via anti‐LILRA5 P4‐11A could induce respiratory burst in neutrophils and monocytes. Anti‐CD89 was used as a positive control as it induces ROS production by cross‐linking FcαR (CD89) and inducing FcRγ signalling [18, 19]. Plate‐bound anti‐CD89 and anti‐LILRA5 P4‐11A, but not isotype IgG1 control, resulted in ROS production by PBMCs (Figure 2F; Figure S3D). As LILRA5 and FcαRI are not expressed on lymphocytes, ROS production is most likely elicited through the monocyte population in the PBMCs. Likewise, anti‐CD89 and anti‐LILRA5 P4‐11A, but not isotype IgG1 control, induced ROS production in neutrophils (Figure 2G; Figure S3E). This data indicates that LILRA5 can induce respiratory burst in human monocytes and neutrophils.

### 2.4 *LILRA5* Transcripts Are Elevated in Systemic Infection and Sepsis

**FIGURE 3:**
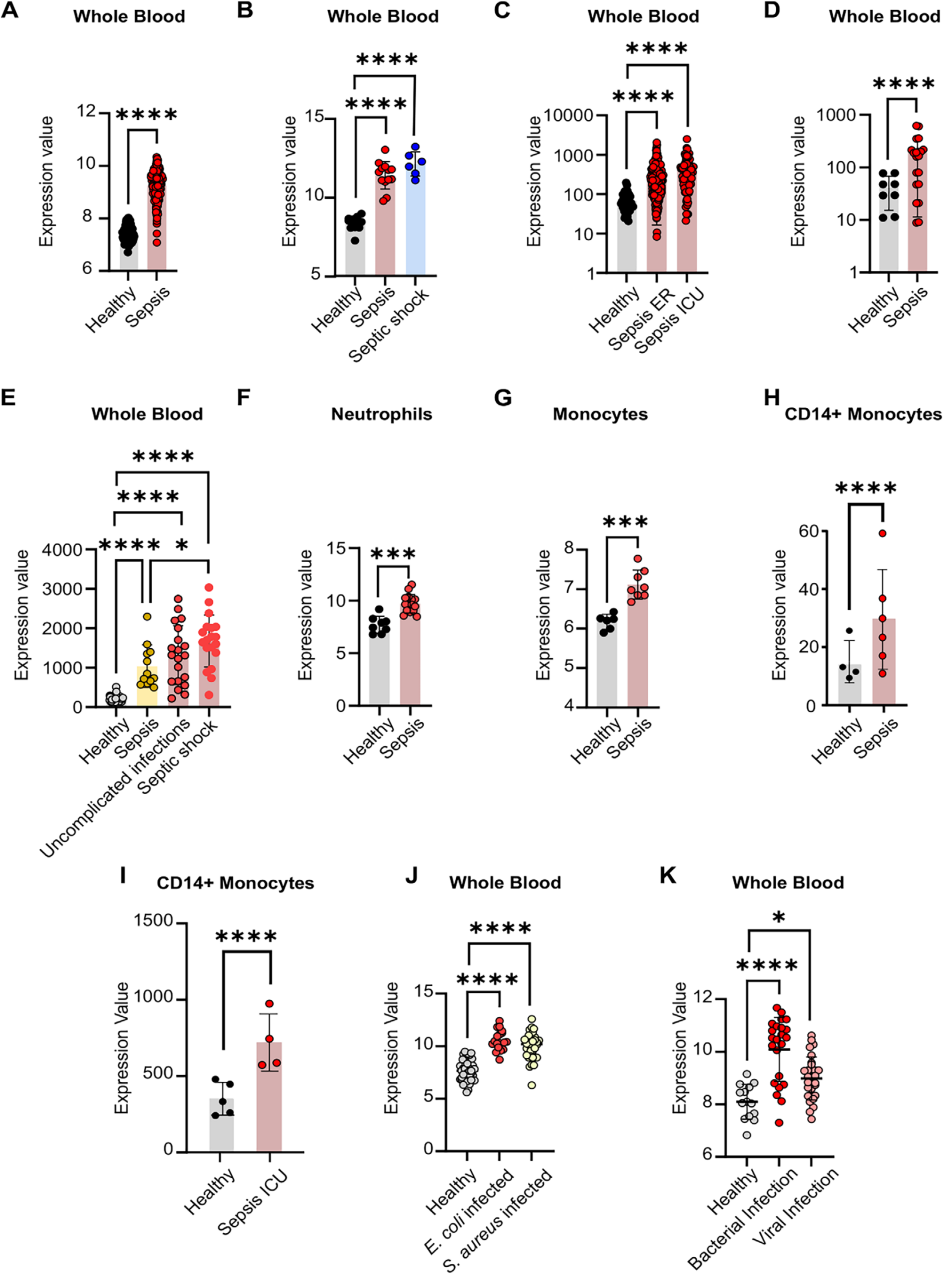
*LILRA5* expression during sepsis and systemic infections. *LILRA5* expression in whole blood of (A) healthy donors (*n* = 83), sepsis patients (*n* = 156; GSE134364). (B) healthy donors (*n* = 12) or sepsis patients (*n* = 13), septic shock patients (*n* = 6; GSE137342). (C) of healthy donors (*n* = 44) or sepsis patients presenting at emergency rooms (ER) (*n* = 266), sepsis patients on intensive care units (ICU; *n* = 82; GSE185263). (D) of healthy donors (*n* = 8), sepsis patients (*n* = 20; GSE232753). (E) of healthy donors (*n* = 40), patients with diagnosed but uncomplicated systemic infection (*n* = 12), sepsis patients (*n* = 20), septic shock patients (*n* = 19; GSE154918). (F) *LILRA5* expression in neutrophils from healthy donors (*n* = 8) or sepsis patients (*n* = 15; GSE64457). LILRA5 expression in CD14+ monocytes from (G) healthy donors (*n* = 6), sepsis patients (*n* = 8; GSE180387), (H) from healthy donors (*n* = 4), sepsis patients (*n* = 6; GSE136200), (I) from healthy donors (*n* = 5), ICU patients with sepsis (*n* = 4; GSE139913). (J) *LILRA5* expression in whole blood from healthy donors (*n* = 14), patients with bacterial infections (*n* = 24), and patients with viral infections (*n* = 28; GSE72810). (K) *LILRA5* expression in whole blood from healthy donors (*n* = 43), patients with *E. coli* infection (*n* = 32), patients with *S. aureus* infection (*n* = 19; GSE33341). In all panels, mean ± SD are shown. Statistics tested by *limma*, where adjusted ****p < 0.0001, ***p < 0.001, and *p <0.05.

As it was recently reported that *LILRA5* expression in monocytes is a biomarker of sepsis [13, 20], we investigated the expression properties of *LILRA5* transcripts and LILRA5 protein in sepsis patients. *LILRA5* expression was elevated in the whole blood of sepsis patients across several datasets (Figure 3A–E). We therefore questioned whether neutrophils and/or monocytes display enhanced *LILRA5* expression. There was enhanced *LILRA5* expression in neutrophils and monocytes from sepsis patients compared with healthy controls (Figure 3F,G). This is further supported by datasets showing significantly enhanced *LILRA5* transcript levels in circulating CD14+ monocytes from critically ill sepsis patients compared with healthy donors (Figure 3H,I). Thus, *LILRA5* expression is increased in whole blood, neutrophils and monocytes during human sepsis.

To understand whether enhanced *LILRA5* expression is a sepsis marker, it is important to establish whether patients with bacterial and viral infections that have not progressed to sepsis display modified *LILRA5* expression. Analysis of the Dix et al. [36] dataset revealed that *LILRA5* expression was increased in the whole blood of patients diagnosed with *S. aureus* or *E. coli* invasive infections (Figure 3J). Likewise, analysis of the Herberg et al. [21] dataset revealed *LILRA5* expression is enhanced in children with confirmed bacterial infection or viral infection compared with healthy controls (Figure 3K). Importantly, analysis of Herwanto et al. [22] indicated that *LILRA5* transcripts in whole blood were significantly increased in patients with diagnosed but uncomplicated infections, in sepsis patients and in septic shock patients compared with healthy controls (Figure 3E). This indicates that *LILRA5* expression is not significantly different between sepsis patients and patients with diagnosed but uncomplicated infections, suggesting that elevated *LILRA5* expression in whole blood is not a marker of sepsis per se.

### 2.5 Surface LILRA5 Expression Does Not Increase Upon Immune Challenge

**FIGURE 4:**
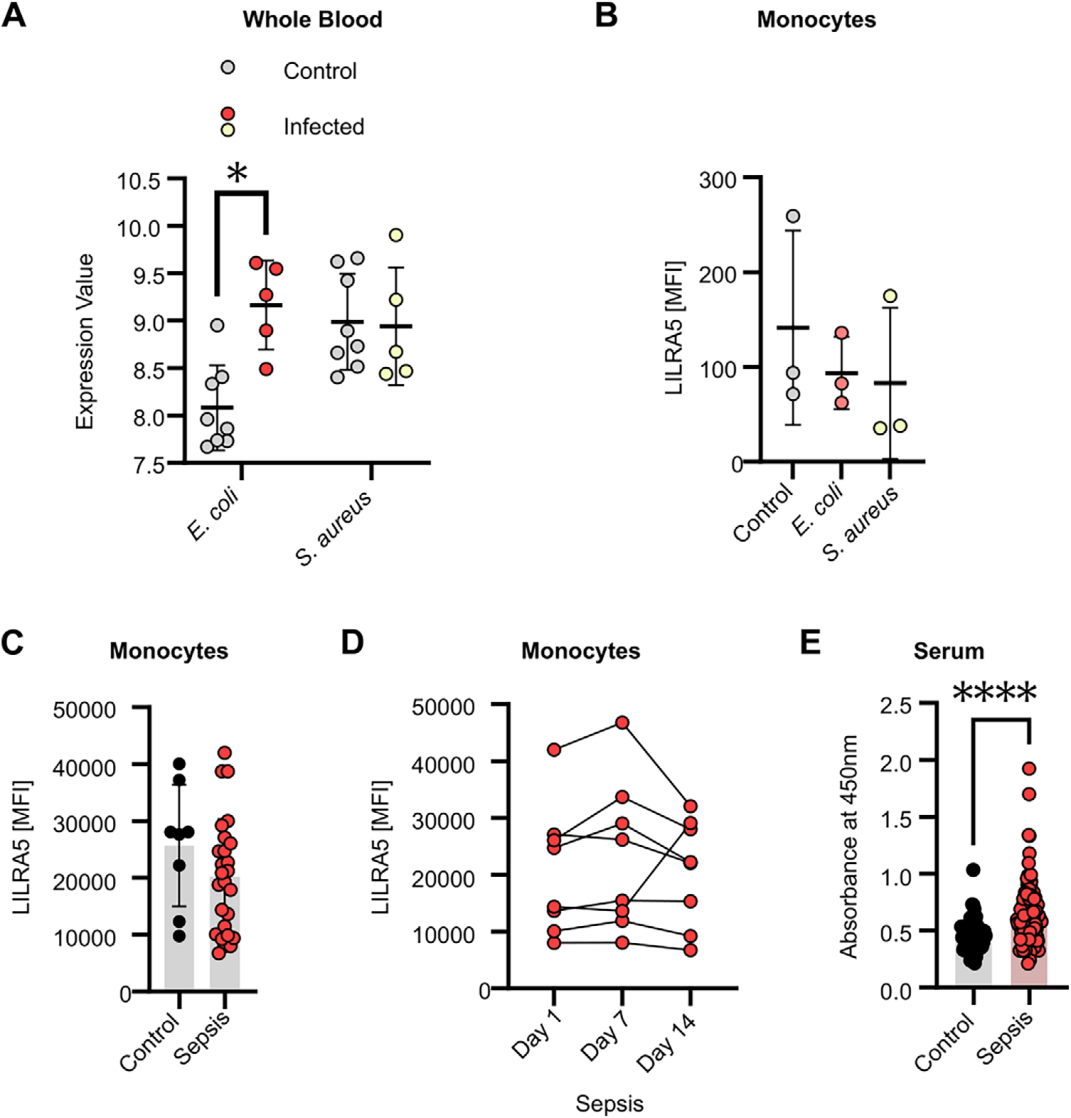
Surface LILRA5 expression does not increase during infection and inflammation. (A) Whole blood *LILRA5* expression after ex vivo infection with *E. coli* (*n* = 5, control *n* = 8) or *S. aureus* (*n* = 5, control *n* = 8; GSE65088). Mean ± SD are shown. Statistics tested by *limma*. (B) Expression of LILRA5 on human monocytes after ex vivo infection of whole blood by *E. coli* or *S. aureus*, from *n* = 3 independent experiments. (C) Surface LILRA5 expression on human monocytes from healthy donors (*n* = 8) and sepsis patients (*n* = 26), upon hospital admission. Mean ± SD are shown. (D) Surface LILRA5 expression on human monocytes from sepsis patients (*n* = 8) at the indicated days since hospital admission. (E) Comparison of sLILRA5 in serum from sepsis patients (*n* = 128) or healthy donors (*n* = 60). Mean ± SD are shown. Student *t*‐test. In all, ****p < 0.0001, ***p < 0.001. **p < 0.01, and *p < 0.05.

Given the elevated *LILRA5* transcripts in whole blood during infectious and inflammatory diseases, we hypothesised that surface LILRA5 expression would be increased on immune cells under these conditions. A previous transcriptome analysis revealed that 4 h of ex vivo infection of human blood with *E. coli*, but not *S. aureus*, significantly increased *LILRA5* expression (Figure 4A). To understand protein expression dynamics, we assessed LILRA5 expression on monocytes purified from whole blood infected ex vivo with *E. coli* or *S. aureus*. Surprisingly, surface LILRA5 expression was not differentially regulated (Figure 4B). To understand whether there was altered surface LILRA5 expression on bacteria‐interacting cells relative to non‐interacting cells, we performed experiments using GFP‐expressing *E. coli* or *S. aureus*. Notably, there was a trend for increased LILRA5 expression on GFP‐positive compared with GFP‐negative cells recovered from *E. coli*‐infected blood and *S. aureus*‐infected whole blood (Figure S4A–C). This data indicates that surface LILRA5 expression is heterogeneous and may be influenced by direct bacterial interaction, rather than solely by the overall presence of bacteria in the bloodstream.

To ascertain whether our ex vivo findings were representative of in vivo immune challenge, we examined the binding of anti‐LILRA5 P4‐11A mAb to PBMCs purified from sepsis patients and healthy controls. The sepsis patients had confirmed infections with Gram‐negative bacteria (*n* = 12; 46%), Gram‐positive bacteria (*n =* 6; 23%), Candida (*n =* 3; 12%), or unidentified aetiology (*n* = 4; 15 %). We specifically measured the LILRA5 signal on monocytes by co‐staining the PBMCs with APC‐conjugated anti‐CD14, PE‐conjugated HLA‐DR and FITC‐conjugated anti‐LILRA5. Notably, there was no significant difference in surface LILRA5 expression levels on monocytes from sepsis patients at day 1 of diagnosis compared with healthy controls (Figure 4C). Furthermore, the surface LILRA5 expression on monocytes remained largely unchanged during the progression of sepsis in individual patients (Figure 4D). Therefore, *LILRA5* transcripts expression in innate immune cells is enhanced during immune challenge, but the surface LILRA5 expression remains unchanged.

### 2.6 Soluble LILRA5 Is Elevated in Serum from Sepsis Patients

Given our previous observations, we queried how *LILRA5* transcripts could increase upon systemic infection and inflammation, but surface LILRA5 expression levels remained unchanged. Alternative mRNA splicing to generate transcripts that encode two different sLILRA5 isoforms could explain these results [9]. Alternatively, it could be explained if surface LILRA5 was shed from cell surfaces. Under either scenario, sLILRA5 levels would be elevated under immune challenge. To test this, we compared sLILRA5 in serum from sepsis patients and healthy donors using the ELISA assay. A quantification of the concentration of sLILRA5 in serum from *n* = 3 sepsis patients and *n* = 3 healthy donors revealed a mean ± SD of 22.2 ± 1.6 ng/mL (Figure S5). Measurement from a larger collection revealed that a significantly higher level of sLILRA5 was present in serum from sepsis patients compared with healthy controls (Figure 4E). Taken together, this suggests that immune challenge increases sLILRA5 levels, indicative of splice changes or cell shedding.

### 2.7 Immune Challenge Impacts LILRA5‐Induced ROS Production

**FIGURE 5:**
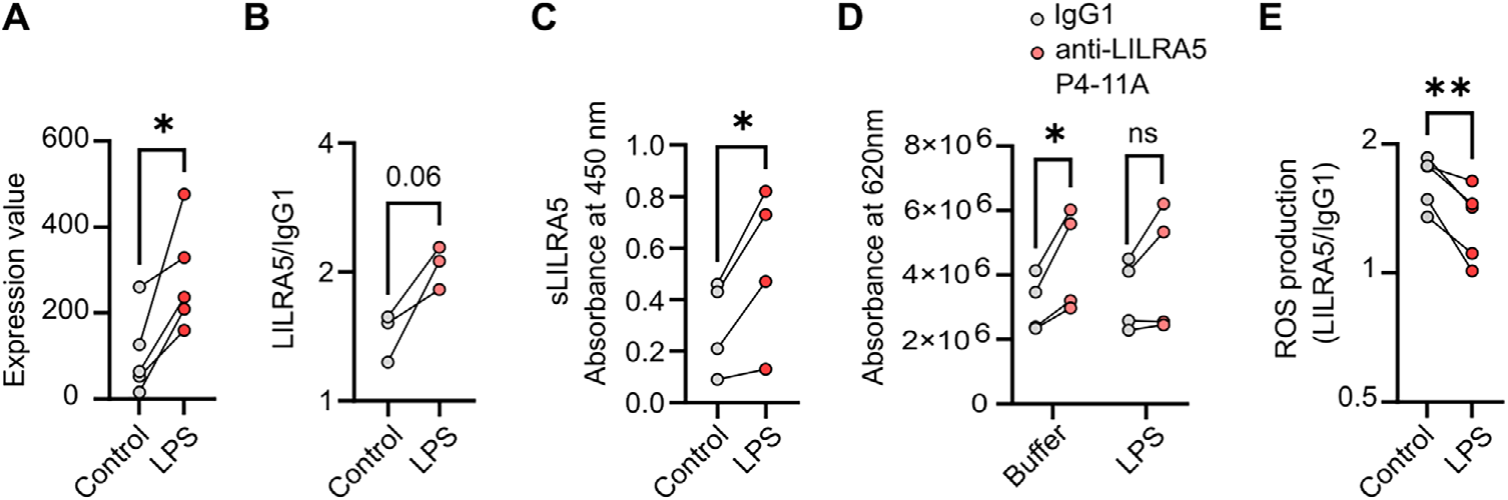
LPS activation reduces LILRA5‐dependent ROS production. (A) *LILRA5* expression by monocytes cultured ± LPS for 18 h (GSE147310). Data from *n* = 5 donors. Statistics tested by *limma*. (B) Surface LILRA5 expression on human monocytes from healthy donors (*n* = 3). Student *t*‐test. (C) sLILRA5 in culture supernatants from PBMCs from healthy donors (*n* = 4), after 18 h culture ± LPS. Student t‐test. (D, E) Production of ROS by PBMCs in response to LILRA5 ± LPS stimulation. Data from *n* = 5 independent donors. Raw ROS production values are shown in (D). Student *t‐*test. The ROS production induced by anti‐LILRA5 P4‐11A relative to IgG1 is compared through area under the curve (AUC), as shown in (E). In all, **p < 0.01, *p < 0.05.

To investigate the functional consequences of altered *LILRA5* transcripts or surface LILRA5 expression, we challenged PBMCs with the potent immune stimulator LPS. LPS induced a significant increase in *LILRA5* transcript expression in monocytes (Figure 5A) and led to a trend (*p* = 0.0683) for increased surface LILRA5 expression (Figure 5B). This correlated with an increased amount of sLILRA5 in the supernatant of cells stimulated with LILRA5 (Figure 5C). Given this, we measured the capacity of anti‐LILRA5 P4‐11A to induce ROS production when cells were pre‐stimulated with LPS. Analysis of raw ROS production values did not identify any statistical difference between cells stimulated with LILRA5 versus LILRA5 + LPS (Figure 5D). However, upon normalisation of the LILRA5/IgG1‐induced ROS production, there was a significant reduction in LILRA5‐dependent ROS production for cells that had been stimulated with LPS compared with untreated cells (Figure 5E). This indicates that LPS challenge increases *LILRA5* transcripts and expression of surface and soluble LILRA5, but that LILRA5 is less effective at inducing respiratory burst.

To further elucidate the functional impact of altered surface LILRA5 levels induced by LPS, we assessed the effects of LILRA5 and/or LPS stimulation on the production of cytokines by PBMCs (Figure S6). We found that stimulation of LILRA5 alone did not alter the production of TNF‐α, IFN‐γ, or IL‐1β, but did significantly reduce IL‐6 production. Notably, LPS challenge significantly increased the production of TNF‐α, IL‐1β, and IL‐6 cytokines, but co‐stimulation of LILRA5 did not significantly affect cytokine production. Taken together, these findings suggest that LPS‐induced changes in LILRA5 expression do not function to modulate cytokine production in PBMCs.

### 2.8 A Putative LILRA5 Ligand in Human Serum

Beyond understanding LILRA5 expression patterns, identifying ligands of LILRA5 is important to determine when LILRA5 drives effective immune responses. Since LILRA5 has no known ligand [10], we tested whether human serum contains putative LILRA5 ligand(s) using the 2B4T chimera NFAT‐GFP reporter cell system. LILRA5CD3ζ‐expressing cells showed a significantly higher percentage of GFP‐positive cells when cultured with serum from healthy controls or sepsis patients, compared with control cells (Figure **S7**). Interestingly, GFP expression in LILRA5CD3ζ‐expressing cells was significantly higher in response to sepsis serum than to healthy serum. These findings suggest the presence of LILRA5 ligands in human serum, with potential upregulation or emergence of a novel ligand during sepsis.

## 3 Discussion

Here, we demonstrate that human phagocytes express surface LILRA5 and that it promotes important effector functions of ROS production and degranulation. Since oxidative burst is induced by immune receptors that act to sense non‐self and that signal through ITAMs [23], our data imply that LILRA5 has a role in mounting innate immune responses. Enhanced ROS production, mediated through a LILRA5‐dependent mechanism during infection or immune challenge, would contribute to the killing of microbes within the phagolysosome following successful phagocytosis. Indeed, deletion of LILRA5 in mice led to enhanced progression of *P. aeruginosa*‐induced inflammation and keratitis [12]. Additionally, the increased release of antimicrobial factors, such as proteases and peptides, into the local environment following LILRA5‐triggered exocytosis of granules, would further promote microbial killing. The activation of LILRA5 on PBMCs has previously been reported to enhance cytokine production [8, 9]. However, we did not observe an increase in the production of TNF‐α, IFN‐γ, IL‐6, or IL‐1β. This could be due to differences in the anti‐LILRA5 monoclonal antibodies used for stimulation, highlighting that different LILRA5 ligands could exert different biological functions.

The level of activating receptor expression on an immune cell is an important determinant of its capacity to induce cellular activation. Notably, a substantially higher expression of surface LILRA5 is present on circulating monocytes compared with neutrophils in healthy individuals, even though there is a comparable level of *LILRA5* transcripts. This differential expression suggests that LILRA5 may have distinct roles in monocytes and neutrophils. Interestingly, even with the lower surface expression of LILRA5 on neutrophils, our assays demonstrate that ROS production can still be effectively induced via LILRA5 cross‐linking. This indicates that LILRA5, although less abundant on neutrophils, is functionally competent and capable of triggering significant immune responses. The higher expression of LILRA5 on the surface of monocytes might reflect a more prominent role in sustained immune activation and regulation, whereas the presence on neutrophils, albeit at lower levels, underscores its importance in rapid, acute responses to infection.

The immune system initiates a swift immune response to detect non‐self to protect the host. The upregulation of *LILRA5* expression at transcript levels in whole blood, monocytes and neutrophils in response to infection or immune challenge suggests that it has a role in sensing or mounting responses against microbes and/or non‐self. In line, it has previously been reported that expression of mRNA encoding membrane‐anchored LILRA5 in monocytes is dynamic and increases upon exposure to cytokines [8]. Given this, we hypothesised that the increased *LILRA5* expression upon immune challenge would be correlated with an increase in surface LILRA5 expression on immune cells. Surprisingly, our study revealed that surface LILRA5 expression does not change on the surface of monocytes during infection with bacterial pathogens ex vivo. Additionally, monocytes from sepsis patients displayed no change in surface LILRA5 expression compared with healthy controls. This could be explained if the increased *LILRA5* transcripts encode sLILRA5 but not membrane‐bound LILRA5. sLILRA5 expression is reported in multiple immune cell types and varies between individuals [24]. In line, sLILRA5 was detected in sera of sepsis patients in our study, in the synovial fluid of rheumatoid arthritis patients, and in sera of patients with liver steatosis [8, 25]. Our studies did identify a trend for increased expression of surface LILRA5 on monocytes after their stimulation with LPS. These results suggest that surface LILRA5 expression is dynamic, changing in response to different stimuli and/or over time. Further investigation is needed to elucidate the precise mechanisms governing both surface and soluble LILRA5 expression, such as the use of long‐read RNA sequencing, and their functional implications in immune responses.

The function of an activating receptor is dependent on the sensing of a ligand, but LILRA5 has no defined ligand [7, 10, 11]. Most activating receptors on phagocytes sense PAMPs, DAMPs, antibody‐opsonised microbes or complement‐opsonised microbes [23, 26]. However, LILRA2, a receptor in the same family, can sense microbially cleaved antibodies to trigger antibacterial effector functions [14]. This raises the prospect that LILRA5 could also sense infection by detecting modified self‐ligands. Our assays with reporter cells indicated that a putative ligand for LILRA5 exists in human serum, as there was an elevated LILRA5 activation signal induced by serum from sepsis patients compared with healthy controls. This could indicate the ligand is in increased abundance in sepsis, or that additional ligands are present under sepsis conditions. Further identifying LILRA5 ligands is critical for advancing our understanding of its biological function. sLILRA5 may act to regulate membrane‐anchored LILRA5 functions by competing for the same ligand.

Enhanced *LILRA5* expression in whole blood or monocytes has been reported as a sepsis biomarker in patient cohorts [12, 13]. However, *LILRA5* transcripts are also increased in patients with diagnosed bloodstream infections. Furthermore, *LILRA5* transcript levels in whole blood do not significantly differ between patients with diagnosed but uncomplicated infection and patients with sepsis. Since levels of *LILRA5* transcripts cannot differentiate these two patient groups, this suggests that *LILRA5* is not a biomarker of sepsis per se. Our analysis of sLILRA5 in serum revealed elevated levels in sepsis patients compared with healthy controls. Additional quantitative studies are required to test the robustness of this finding and to ascertain whether quantifying sLILRA5 in sera is a putative biomarker of sepsis.

In conclusion, this study was underpinned by the development of a novel LILRA5‐targeting mAb that detects LILRA5 with high specificity and possesses agonistic properties. LILRA5 is expressed by human phagocytes and promotes ROS production. Immune challenge can induce dynamic increases in *LILRA5* transcript levels, but unchanged or increased surface LILRA5 expression. This regulates LILRA5‐dependent ROS production. Future studies are required to identify LILRA5 ligands and to further elucidate the regulatory mechanisms and functions of membrane‐bound LILRA5 and sLILRA5.

## 4 Data Limitations and Perspectives

We acknowledge that the present study is subject to certain limitations. These include the use of the 2B4 NFAT‐GFP T cells as a reporter system for identifying receptor ligands. This system has proven effective for forcing LILR receptors to signal through a well‐defined pathway and readout. However, it is an artificial system, as LILRA5 typically associates with the endogenous FcRγ. Future studies could develop myeloid cell‐based reporter systems for LILRs to provide a more physiologically relevant assessment of ligand functions. Additionally, there are limitations to our analysis of *LILRA5* expression. Whilst our analysis of overall mRNA reveals changes in transcript levels, this analysis does not capture the expression of different isoforms arising from alternative splicing. Future investigations employing more advanced techniques, such as long‐read RNA sequencing, could provide a more in‐depth analysis of the LILRA5 transcript and unravel the role of changes in splice variants.

## 5 Materials and Methods

### 5.1 Expression and Purification of rLILR‐His

EXPI293F cells were cultured as described [19, 27]. To construct expression vectors for recombinant (r)LILRA5, rLILR and rLAIR‐1, the signal peptide and extracellular domains of LILRs were amplified from cDNA vectors (Table S1) and inserted into pcDNA3.4 vectors as described [19]. Recombinant His‐tagged proteins were purified as described [19].

### 5.2 Generation of anti‐LILRA5 mAb

Generation of LILRA5‐specific mAb was performed using the hybridoma technique of Köhler and Milstein [19]. A BALB/c mouse was immunised with rLILRA5‐His (David's Biotechnology, Germany). Spleen cells from BALB/c mice were recovered and fused to myeloma NS1/0 cells. Soluble IgG expressed by individual hybridoma clones was tested for binding rLILRA5‐His by ELISA, allowing the identification of clone P4‐11A. The mAb was produced in a serum‐ and azide‐free medium.

### 5.3 Leukocyte Immunophenotyping by Flow Cytometry

Hetasep (STEMCELL Technologies) was diluted in whole blood (1:7 v/v) and incubated for 30 min at room temperature. The supernatant was collected and washed with RPMI1640 with stable glutamine (Bio&Sell) supplemented with 2% FCS (Biochrom; v/v) and penicillin/streptomycin (Gibco). Platelets were removed by centrifugation at 200 g for 10 min without a brake. Residual red blood cells were lysed with Red Blood Cell Lysis Solution (Miltenyi Biotec). Isolated leukocytes were incubated with human TruStain FcX (Biolegend) and stained with 10 µg anti‐CD14‐APC (Immunotools), anti‐CEACAM8 (clone 6/40c) and anti‐LILRA5‐11A (in‐house labelled with PE). Staining with the corresponding isotype controls (mIgG‐FITC, mIgG‐PE, mIgG‐APC; Immunotools) was performed to determine the threshold for specific staining. Data were acquired using MACSQuant Analyzer 10 (Miltenyi Biotec) and analysed using MACSQuantify Software 2.13 (Miltenyi Biotec).

### 5.4 Isolation of Primary Human Neutrophils and PBMCs

Neutrophils and PBMCs were isolated from whole blood by Ficoll/Histopaque centrifugation and resuspended in RPMI1640 supplemented with 5% FCS, as previously described [19]. Neutrophils were isolated with >98% purity and 99% viability.

### 5.5 Binding of mAb to Magnetic Beads or Cells

Binding of antibodies to magnetic beads (Dynabeads, ThermoFisher) coated with C‐terminal His‐tagged rLILR or rLAIR‐1 proteins was performed as previously described [19]. For cell assays, 45 µL of 5 × 10⁶ cells/mL of cells were incubated with 5 µg/mL of mAb. After washing, cells were incubated with PE‐conjugated goat anti‐mouse IgG (1:300; Invitrogen). The fluorescence of cells was measured by flow cytometry.

### 5.6 ELISA for Detection of sLILRA5

Wells of 96‐well plates were coated overnight with 50 µL of 2 µg/mL anti‐LILRA5 clone P4‐11A in PBS. After blocking, wells were incubated with 100 µL of rLILRA5‐His or rLAIR1‐His in buffer or heat‐inactivated human serum samples for 1 h at 4°C. For the detection of LILRA5, rabbit anti‐LILRA5 pAb (1:2000; Sino Biological) was incubated as above. For signal detection, horseradish peroxidase (HRP)‐conjugated goat anti‐rabbit‐IgG (1:10,000; Life Technologies) was incubated as above. The assay was developed with 1xTMB substrate and Stop solution for TMB substrate (Invitrogen). All washes with PBS + 0.05% Tween‐20.

### 5.7 Generation of LILRA5‐Expressing U937 Cell Line

U937 cell lines were cultured as previously described [19]. To construct LILRA5‐expressing U937 cells, the coding domain sequence of *LILRA5* was amplified from a LILRA5 cDNA vector (HG16059‐G, BioConnect) using primers 5′‐GAGCTAGCAGTATTAATTAACCACCATGGCACCATGGTCTCATCCATC‐3′ and 5′‐GTACCGGTTAGGATGCATGCTCACCTTCCAGCTGCAGCTTGGG‐3′. PCR amplification was performed using Phusion High‐Fidelity Taq Polymerase (Thermo Fisher Scientific) and thermocycling as follows: 1 cycle (98°C for 2 min), 35 cycles (98°C 15 s, 60°C 30 s, and 72°C 30 s), and 1 cycle (72°C 10 min). The purified amplicon was ligated into a dual promoter lentiviral vector (BIC‐PGK‐Zeo‐T2a‐mAmetrine; RP172 derived from no.2025.pCCLsin.PPT.pA.CTE.4⋅‐scrT.eGFP.mCMV.hPGK.NG‐FR.pre as previously described [19]. Lentiviral particles were created and transfected into U937 cells as previously described [19].

### 5.8 Generation of LILRA5CD3ζ Reporter Cell Line and GFP Induction

2B4 NFAT‐GFP T cell reporter cells were cultured as described [19]. Assays were performed as described [19]. A LILRA5CD3ζ reporter cell line was generated as described [19]. In brief, the coding domain sequence of the *LILRA5* extracellular and transmembrane domains and the CD3ζ cytoplasmic tail was synthesised by Integrated DNA Technologies and ligated into the RP172 vector. After transduction into 2B4 NFAT‐GFP T cells, the selected transductant cells were tested for surface LILRA5 expression using anti‐LILRA5 pAb (16059‐RP01; Sino Biological) and flow cytometry analysis. TC‐treated 96‐well plates were coated with 5 µg/mL Hamster anti‐mouse‐CD3 (145‐2C11, BioRad), anti‐LILRA5 pAb (16059‐RP01; Sino Biological), anti‐LILRA5 P4‐11A, or IgG isotype diluted in PBS.

### 5.9 Reactive Oxygen Species (ROS) Production

White 96‐well plates were coated with 50 µl of 5 µg/ml anti‐LILRA5 clone P4‐11A, anti‐CD89 (MCA1824; Bio Rad) or IgG1 Isotype (0102‐01; Southern Biotech) diluted in NaHCO_3_ buffer, pH8.6. Neutrophils and PBMCs were washed with ice‐cold reaction buffer (20 mM HEPES, 140 mM NaCl, 1 mM CaCl_2_, 5 mM Glucose, pH 7.4) three times. To each well, 100 µL of 1 × 10⁶ cells and 100 µL of reaction buffer containing 20 mM Amplex Red (Life Technologies) were added. For PBMC assays, the reaction buffer was supplemented with 10 µL of horseradish peroxidase (Sigma Aldrich). Fluorescence was measured by a CLARIOstar microplate reader for 2 h. Area under the curve (AUC) for each stimulus was calculated after subtraction of background/PBS ROS production. In specific experiments, cells were stimulated with 5 µg/ml LPS from *Escherichia coli* O111:B4 (Sigma–Aldrich) for one hour at 37°C with 5% CO_2_.

### 5.10 Activation of Neutrophils and Monocytes by LILRA5 Cross‐Linking and/or LPS

96‐well plates were coated with 50 µL of 5 µg/mL anti‐LILRA5 clone P4‐11A or IgG1 Isotype control (0102‐01; Southern Biotech). Neutrophils and PBMCs were resuspended to 1 × 10⁶ cells/mL in a solution made up of 80% RPMI 1640 + penicillin/streptomycin and 20% plasma from the same donor. To each well, 200 µL of cells was added and cultured for 24 h at 37°C with 5% CO_2_. In certain assays, neutrophils and PBMCs were stimulated with 1 µg/mL or 10 ng/mL LPS from *E. coli* O111:B4 (Sigma–Aldrich) for 24 h at 37°C with 5% CO_2_, respectively. Neutrophils were stained with anti‐CD66b‐FITC (1:50; BD Biosciences). PBMCs were stained with 3 µg/mL anti‐CD14‐FITC (367116, BioLegend). The fluorescence of cells was measured by flow cytometry and analysed based on the forward‐ and side‐scatter plots.

### 5.11 *LILRA5* Expression Profiling in Transcriptome Studies

Retrieved data on Gene Expression Omnibus (GEO–NCBI) using GEO2R (NCBI) were used to measure *LILRA5* expression in (1) whole blood of sepsis patients and controls = GSE134364 [29], GSE137342 from Mukhopadhyay, GSE185263 [30], (2) neutrophils from sepsis patients and controls = GSE64457 [31], (3) in monocytes from sepsis patients and controls = GSE180387 [32], GSE136200 [33], GSE139913 [34], (4) in whole blood of sepsis patients, septic shock patients, patients with diagnosed but uncomplicated infections and controls GSE154918 [22], (5) in whole blood of patients with diagnosed bacterial or viral infections = GSE72810 [21] and GSE33341 [35], (6) in whole blood after ex vivo infection with *E. coli* or *S. aureus* = GSE65088 [36], (7) in monocytes after culture for 18 h ± LPS = GSE147310 [37].

### 5.12 LPS‐stimulation of Leukocytes and Quantification of Surface LILRA5 and sLILRA5

Leukocytes were cultured in RPMI1640 supplemented with 4% FCS at 4 × 10^5^ cells/well/200 µL in 96‐well ultra‐low attachment culture plates (Corning) at 37°C with 5% CO_2_. After 60 min, cells were stimulated with 50 ng/ml *E. coli* 026:B6 LPS (Sigma) for 18 h. Leukocytes were incubated with human TruStain FcX (BioLegend) and stained with anti‐CD14‐APC (Immunotools), anti‐HLADR‐PE (Invitrogen), and anti‐LILRA5‐11A (in‐house labelled with FITC) as described previously [38]. Staining with the corresponding isotype control antibodies was performed to determine the threshold for specific staining. The threshold was set at 3% false‐positive cells. Data were acquired using CytoFLEX S (Beckman Coulter) and analysed using CytExpert (Beckman Coulter). Monocytes were gated as CD14+HLA‐DR+ cells, and the median fluorescence intensity (MFI) of LILRA5 or isotype control was determined. Individual expression of LILRA5 was calculated as [MFI LILRA5 ‐ MFI Isotype control]. Cell‐free supernatants were collected for measurement of TNFα, IL‐1β, IL‐6 and IFN‐γ, according to the manufacturer's instructions (PeproTech).

### 5.13 Whole Blood Infection and Measurement of Surface LILRA5 Expression on Monocytes

1 × 10⁷ of *E. coli* MG1655, *E. coli* TOP10 + pULtra.GFP‐Kanamycin, or *S. aureus* JE2 or *S. aureus* Wood‐GFP were inoculated into 10 mL of whole blood for 2 h at 37°C. PBMCs were then isolated as above. 45 µL of 5×10⁶ cells/mL were incubated with 3 µg/mL IgG1 isotype (0102‐01; Southern Biotech), PE‐conjugated IgG1 Isotype control, or anti‐LILRA5 P4‐11A mAb or PE‐conjugated anti‐LILRA5 P4‐11A mAb for 30 min at 4°C. Secondary detection was performed with PE‐conjugated goat anti‐mouse IgG (1:300; Invitrogen) if required. The fluorescence of cells was measured by flow cytometry.

### 5.14 Analysis of Monocytes and Serum from Sepsis Patients

Sepsis was diagnosed according to the Sepsis‐3 criteria [39]. *n* = 26 patients (16 males and 10 females) with sepsis were enrolled at the Department of Anesthesiology and Intensive Care Medicine, University Hospital Jena, Germany. The median (IQ range) age of the patients was 65 (53‐72). The source of infection was Gram‐negative bacteria (*n* = 12; 46%), Gram‐positive bacteria (*n =* 6; 23%), Candida (*n =* 3; 12%), or unidentified (4; 15%). Heparinised blood was drawn within 24 h after admission to the intensive care unit (ICU). If the patient remained on the ICU, blood was collected up to two more times on day 7 and day 14 after admission. Whole blood from healthy donors (*n* = 9) with a median age (IQ range) of 52 (30–57) was used as a control.

## Author Contributions

Zuyi Fu, Matevž Rumpret, Irina Kube‐Golovin, Mykola Lyndin, Vera Solntceva, Yuxi Zhao, and Anastasia Konieva performed experiments. Zuyi Fu, Matevž Rumpret, Irina Kube‐Golovin, and Alex J. McCarthy analysed data, visualised data, and wrote the original draft. Na Liu, Adrian T. Press, Stefanie B. Flohé and Gunther Wennemuth provided scientific help and critically reviewed the manuscript. Alex J. McCarthy, Irina Kube‐Golovin, and Bernhard B. Singer conceptualised and directed the study. Alex J. McCarthy supervised the analysis and interpretation of the data. All authors contributed to the article and approved the submitted version.

## Ethics Approval Statement

Human blood was obtained from healthy donors, approved by the Regional Ethics Committee and Imperial College Healthcare NHS Trust Tissue Bank (Regional Ethics Committee approval no. 17/WA/0161, Imperial College Healthcare Tissue Bank Human Tissue Authority licence no. 12275, and Imperial College Research Ethics Committee no. 19IC5166). Samples from sepsis patients and controls were approved by the local ethics committee of the Friedrich‐Schiller‐University Jena (ID:2019‐1306_2‐Material and ID:2022‐2847‐Material). Serum sepsis samples were approved by the Ethics Committee of Sumy State University (protocol number 12, approval date December 11th 2021).

## Conflicts of Interest

The authors declare no conflicts of interest.

## Peer Review

The peer review history for this article is available at https://publons.com/publon/10.1002/eji.70079.

## Patient Consent Statement

All samples were collected after receiving signed informed consent from all participants.

## Supporting information




**Supporting Information File 1**: eji70079‐sup‐0001‐SuppMat.pdf

## Data Availability

The data that support the findings of this study are available from the corresponding author upon reasonable request.
