# Supplementary material for: LILRA5 Functions to Induce ROS Production on Innate Immune Cells
Source: Eur J Immunol. 2025 Oct 20;55(10):e70079. doi: 10.1002/eji.70079 (PMC12537994; doi:10.1002/eji.70079)
Supplement: Supplementary file 1 — Supporting Information File 1: eji70079‐sup‐0001‐SuppMat.pdf [file EJI-55-e70079-s001.pdf]

Supporting Information

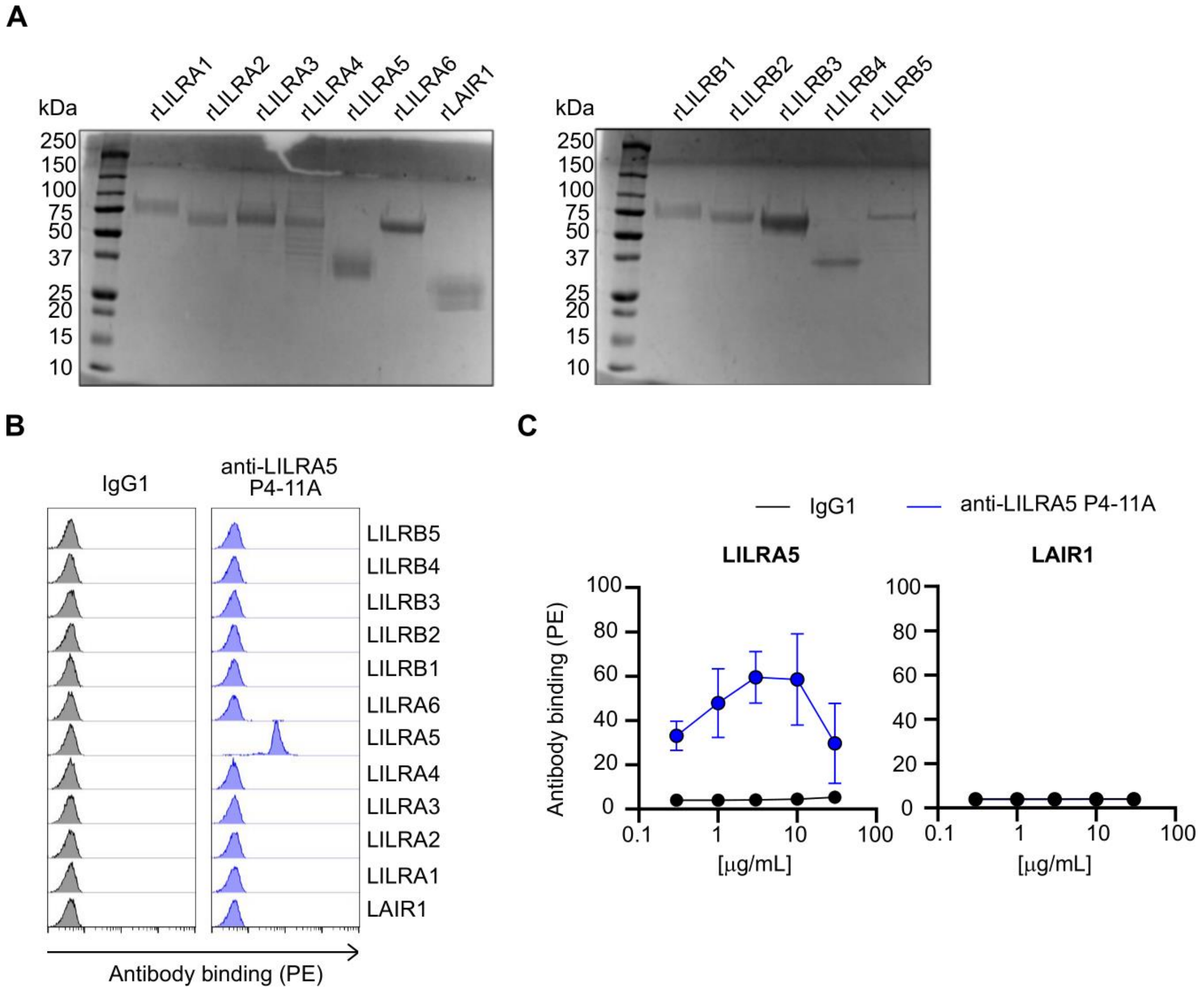

Figure S1: anti-LILRA5 P4-11A mAb binds to LILRA5 with high-specificity. (A) SDS-PAGE analysis of recombinant (r) proteins, used for assessing the specificity of anti-LILRA5 P4-11A mAb. **(B)** Specificity of anti-LILRA5 P4-11A antibody for LILRA5. Magnetic beads coated with rLILR or control proteins and analysed for binding of anti-LILRA5 P4-11A mAb. mAb binding was detected using anti-IgG mAb and flow cytometric analysis. *n* = 3 from 3 independent experiments, one representative experiment is shown. **(C)** Concentration-dependent binding of anti-LILRA5 P4-11A mAb to rLILRA5-coated magnetic beads. mAb binding was detected using anti-IgG mAb and flow cytometric analysis. *n* = 3 from 3 independent experiments.

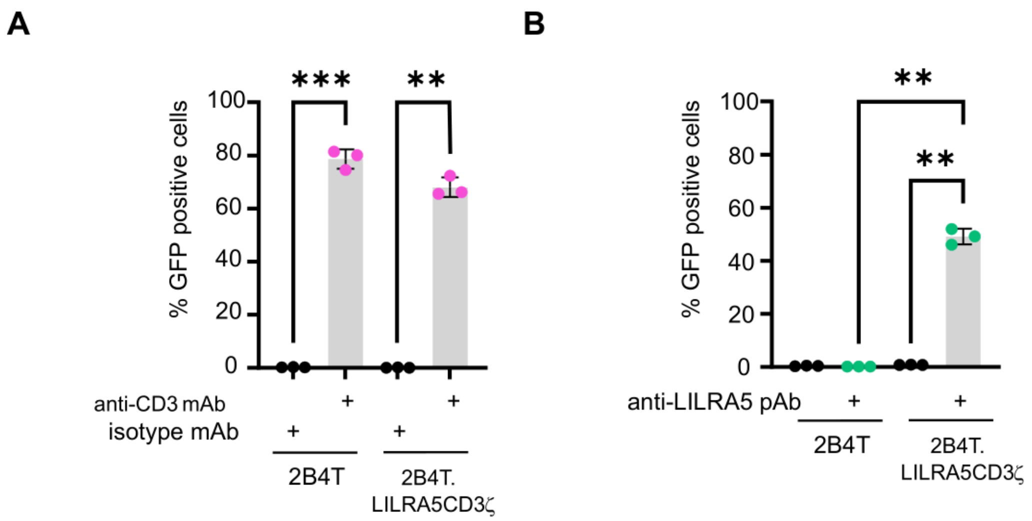

Figure S2: Validation of LILRA5. (A) Cross-linking capacity of plate-bound anti-CD3 mAb to induce GFP expression in the LILRA5CD3ζ reporter 2B4T cell line and control 2B4T cell line. GFP expression by LILRA5CD3ζ+ 2B4T cells or control 2B4T cells was assessed by flow cytometric analysis after incubation in wells containing plate-bound anti-CD3 or isotype mAb. The % of GFP+ cells was quantified. Mean ± SD of $n = 3$ independent experiments are shown. One-way ANOVA, where ***$p < 0.001$, **$p < 0.01$. **(B)** Cross-linking capacity of plate-bound anti-LILRA5 pAb to induce GFP expression in the LILRA5CD3ζ reporter 2B4T cell line and control 2B4T cell line. GFP expression by LILRA5CD3ζ+ 2B4T cells or control 2B4T cells was assessed by flow cytometric analysis after incubation in wells containing plate-bound anti-LILRA5 or buffer control. The % of GFP+ cells were quantified. Mean ± SD of $n = 3$ independent experiments are shown. One-way ANOVA, where ***$p < 0.001$, **$p < 0.01$.

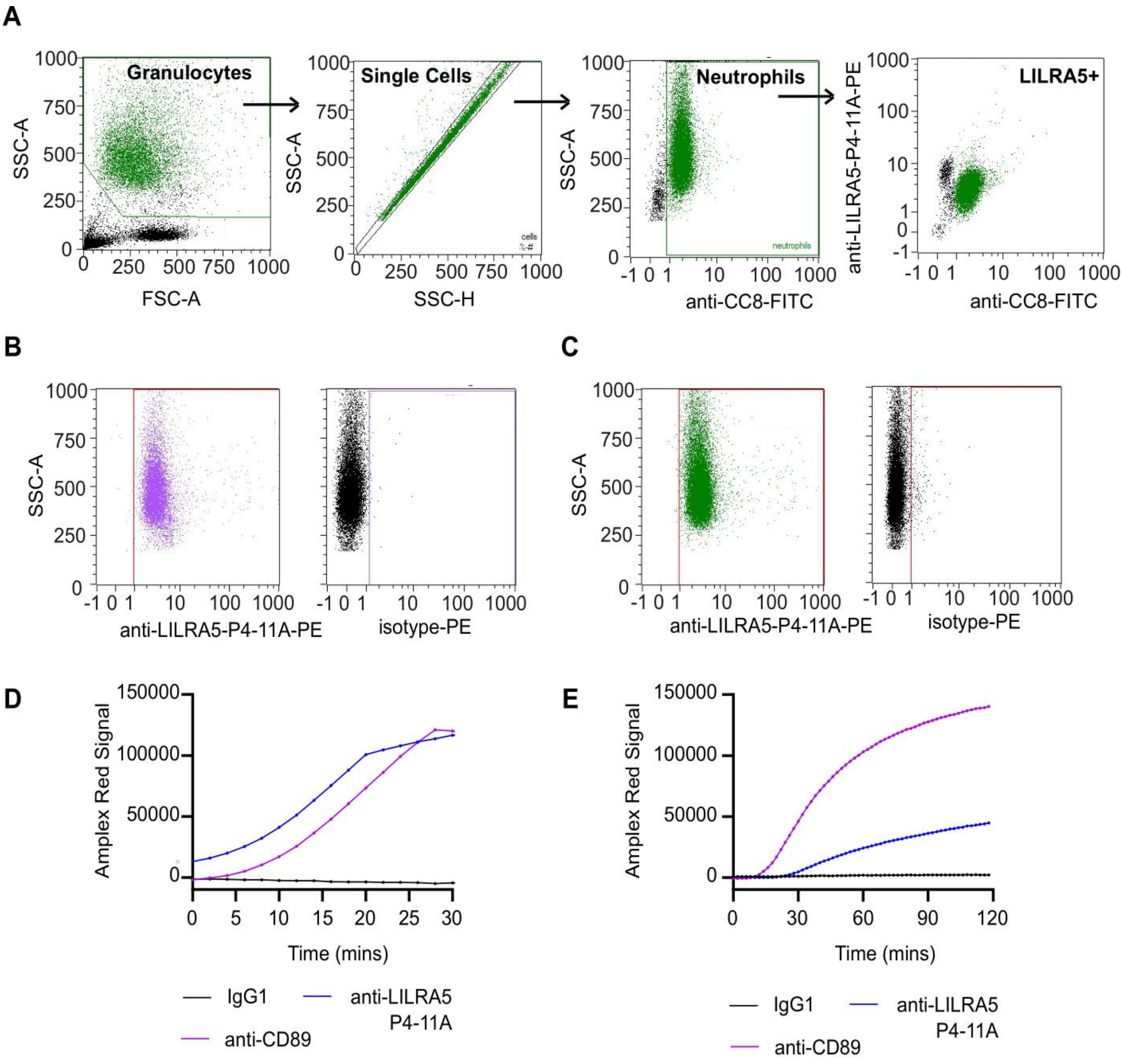

Figure S3: Gating strategy and expression of LILRA5. (A) Expression of LILRA5 on neutrophils (CEACAM8-positive). Representative example showing the gating strategy used. **(B)** Representative flow cytometry histogram showing LILRA5 expression on CD14+ monocytes. **(C)** Representative flow cytometry histogram showing LILRA5 expression on CEACAM8+ neutrophils. **(D** and **E)** Stimulating LILRA5 or CD89 on (D) PBMCs and (E) neutrophils induces reactive oxygen species (ROS) production, as measured using Amplex Red. Representative plots are shown.

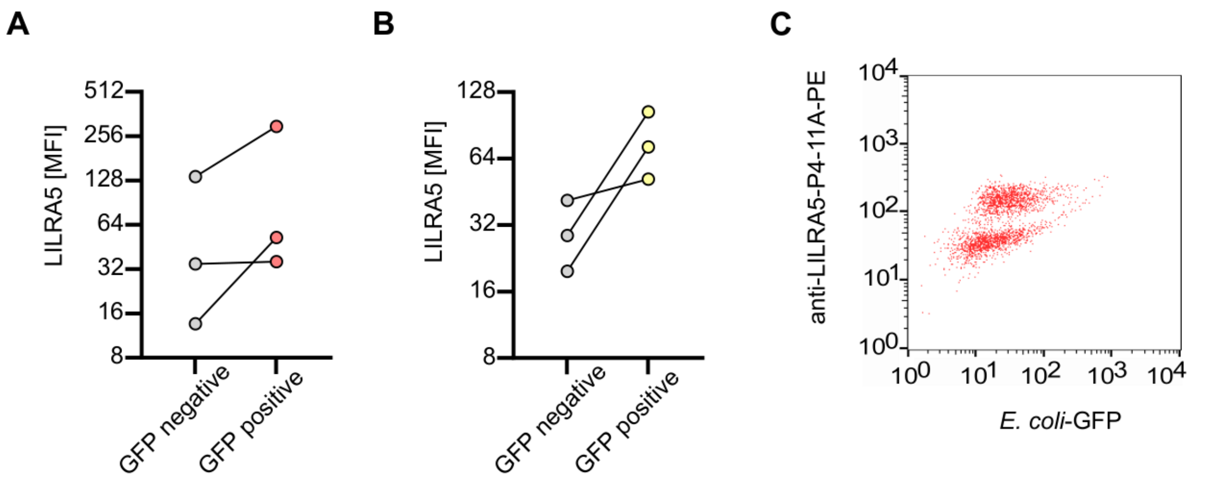

Figure S4: Surface LILRA5 expression is significantly reduced on bacterial interacting and non-interacting immune cells. Expression of LILRA5 on human monocytes (n = 3) following *ex vivo* whole blood infection with **(A)** GFP-expressing *E. coli* and **(B)** GFP-expressing *S. aureus*. Signal was measured by flow cytometry and analysed using One-way ANOVA to compare the mean fluorescence intensity (MFI) of anti-LILRA5 P4-11A mAb (intensity subtracted from isotype control) on uninfected cells, GFP-negative (extracellularly infected) cells, and GFP-positive (intracellularly infected) cells. Data are presented as mean ± SD, where $*p < 0.05$. **(C)** Representative flow cytometry plot showing LILRA5-expression on the surface of monocytes recovered from whole human blood after infection with GFP-expressing *E. coli*.

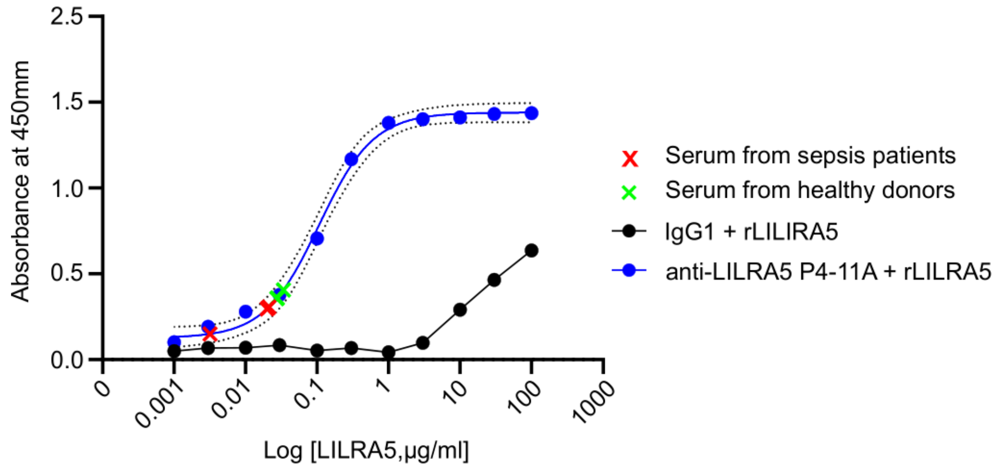

Figure S5: Quantification of sLILRA5 in serum from healthy donors and sepsis patients. sLILRA5 levels in serum from healthy individuals (*n* = 3) and sepsis patients (*n* = 3) were measured by ELISA. Anti-LILRA5 P4-11A monoclonal antibody or an isotype control was used as the capture antibody. A titration curve of recombinant LILRA5-His (*n* = 3), detected using the P4-11A antibody is also shown.

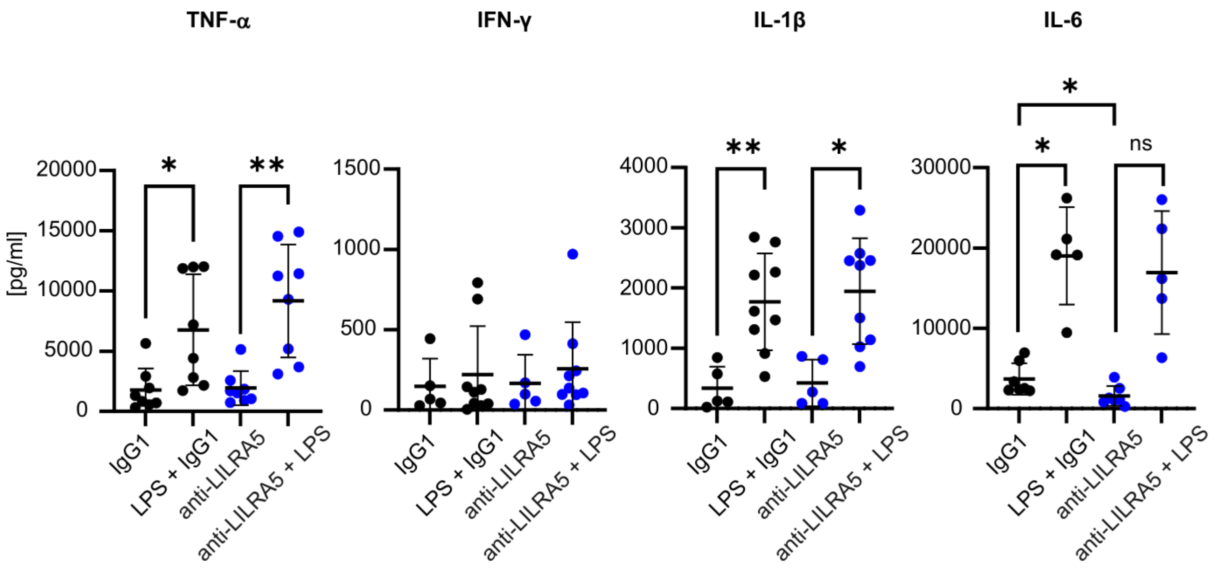

Figure S6: Cytokine production in response to LILRA5 +/- LPS stimulation. Quantification of TNF-α, IFN-γ, IL-1β and IL-6 cytokine production by PBMCs in response to LILRA5 ± LPS stimulation, measured by ELISA after 24 hours. Data were analysed using one-way ANOVA and are presented as mean ± SD, where *$p < 0.05$ and **$p < 0.01$.

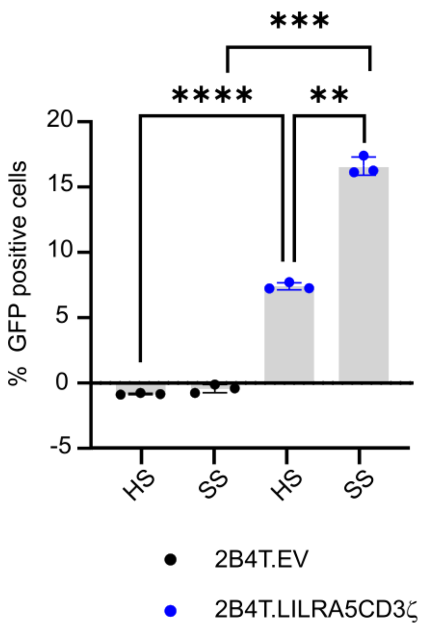

Figure S7. Potential LILRA5 ligands in serum from healthy donors and sepsis patients. Identification of potential LILRA5 ligands using a LILRA5CD3ζ reporter 2B4 T cell line. GFP expression in LILRA5CD3ζ reporter 2B4 T cells or control 2B4 T cells was measured by flow cytometry after overnight incubation with serum from healthy donors (HS; n = 3) or sepsis patients (SS; n = 3). The percentage of GFP-positive cells was quantified. Data are presented as mean ± SD, analysed using one-way ANOVA, where ** $p < 0.01$, *** $p < 0.001$, **** $p < 0.0001$.

Table S1: cDNA vectors and amplification primers

cDNA vector	Primers (5' to 3')
LILRA1<br>HG17220-G (BioConnect)	Forward:<br>CTCTAGAGGATCGAACCCTTGGATCCACCACCATGACCCCCATC GTCACAGTCCTG<br><br>Reverse:<br>CTAACCGGTAGGGATCGAACCCTTGCGGCCGCCTAGTGGTGAT GGTGGTGATGATTCTCCACTGTGTAATCCTGGGGG
LILRA2<br>HG13273-G (BioConnect)	Forward:<br>CTCTAGAGGATCGAACCCTTGGATCCACCACCATGACCCCCATC CTCACGGTCCTG<br><br>Reverse:<br>CTAACCGGTAGGGATCGAACCCTTGCGGCCGCCTACTAGTGGT GATGGTGGTGATGTGTGTAATCCTGGGGGTGTTGGC
LILRA3<br>HG13549-G (BioConnect)	Forward:<br>CTCTAGAGGATCGAACCCTTGAATTCACCACCATGATGACCTCC ATCCTCACGGTC<br><br>Reverse:<br>CTAACCGGTAGGGATCGAACCCTTGCGGCCGCCTACTAGTGGT GATGGTGGTGATGCTCACCAGCCTTGGAGTCGGACT
LILRA4<br>HG16058-G (BioConnect)	Forward:<br>CTAACCGGTAGGGATCGAACCCTTGCGGCCGCCTACTAGTGGT GATGGTGGTGATGCTCACCAGCCTTGGAGTCGGACT<br><br>Reverse:<br>CTAACCGGTAGGGATCGAACCCTTGCGGCCGCCTAGTGGTGAT GGTGGTGATGATTCTCCACTGTGTAATCCTTGG
LILRA5<br>HG16059-G (BioConnect)	Forward:<br>CTCTAGAGGATCGAACCCTTGGATCCACCACCATGGCACCATG GTCTCATCCATC<br><br>Reverse:<br>CTAACCGGTAGGGATCGAACCCTTGCGGCCGCCTAGTGGTGAT

	GGTGGTGATGGCGGATGAGATTCTCTACTGCG
LILRB2<br>HG14132-G (BioConnect)	Forward:<br>CTCTAGAGGATCGAACCCTTGGATCCACCACCATGACCCCCATC<br>GTCACAGTCCTG<br><br>Reverse:<br>CTAACCGGTAGGGATCGAACCCTTGCGGCCGCCTAGTGGTGAT<br>GGTGGTGATGCGACCCAGTGGGGGTGAGGGGC
LILRB4<br>HG16742-G (BioConnect)	Forward:<br>CTCTAGAGGATCGAACCCTTGAATTCACCACCATGATCCCCACC<br>TTCACGGCTCTG<br><br>Reverse:<br>CTAACCGGTAGGGATCGAACCCTTGCGGCCGCCTAGTGGTGAT<br>GGTGGTGATGCTCCCAGTCGGTTCTCAGACC
LILRB5<br>HG17221-G (BioConnect)	Forward:<br>CTCTAGAGGATCGAACCCTTGGATCCACCACCATGACCCTCACC<br>CTCTCAGTCCTG<br><br>Reverse:<br>CTAACCGGTAGGGATCGAACCCTTGCGGCCGCCTAGTGGTGAT<br>GGTGGTGATGCCCCAGGTGCCTTCCCAGACC
